# Fatty acids do not pay the toll: effect of SFA and PUFA on human adipose tissue and mature adipocytes inflammation

**DOI:** 10.1186/1476-511X-11-175

**Published:** 2012-12-21

**Authors:** Ravi Kumar Murumalla, Manoj Kumar Gunasekaran, Jibesh Kumar Padhan, Karima Bencharif, Lydie Gence, Franck Festy, Maya Césari, Régis Roche, Laurence Hoareau

**Affiliations:** 1 GEICO (Study Group on Chronic Inflammation and Obesity), Platform ‘Cyclotron Reunion Ocean Indien’ CYROI, 2, rue Maxime Rivière, Sainte-Clotilde, Reunion Island, 97 490, France

**Keywords:** FFA, Human adipose tissue, Inflammation, TLR

## Abstract

**Background:**

On the basis that high fat diet induces inflammation in adipose tissue, we wanted to test the effect of dietary saturated and polysunsaturated fatty acids on human adipose tissue and adipocytes inflammation. Moreover we wanted to determine if TLR2 and TLR4 are involved in this pathway.

**Methods:**

Human adipose tissue and adipocytes primary cultures were treated with endotoxin-free BSA conjugated with SFA (lauric acid and palmitic acid - LA and PA) and PUFA (eicosapentaeneic acid, docosahexaenoic acid and oleic acid - EPA, DHA and OA) with or without LPS. Cytokines were then assayed by ELISA (TNF-alpha, IL-6 and MCP-1). In order to determine if TLR2 and TLR4 are activated by fatty acid (FA), we used HEK-Blue cells transfected by genes from TLR2 or TLR4 pathways associated with secreted alkaline phosphatase reporter gene.

**Results:**

None of the FA tested in HEK-Blue cells were able to activate TLR2 or TLR4, which is concordant with the fact that after FA treatment, adipose tissue and adipocytes cytokines levels remain the same as controls. However, all the PUFA tested: DHA, EPA and to a lesser extent OA down-regulated TNF-alpha, IL-6 and MCP-1 secretion in human adipose tissue and adipocytes cultures.

**Conclusions:**

This study first confirms that FA do not activate TLR2 and TLR4. Moreover by using endotoxin-free BSA, both SFA and PUFA tested were not proinflammatory in human adipose tissue and adipocytes model. More interestingly we showed that some PUFA exert an anti-inflammatory action in human adipose tissue and adipocytes model. These results are important since they clarify the relationship between dietary fatty acids and inflammation linked to obesity.

## Background

Recent decades, overnutrition-related diseases are increasing in developed countries due to inappropriate diets. Indeed high fat diet regulates some metabolic dysfunctions which lead to low grade inflammation, long before the onset of overweight and obesity [1]. Specifically it has been shown that high fat diet leads to hypertrophy and inflammation in adipocytes [2,3], which release proinflammatory cytokines and free fatty acids *via* lipolysis [4]. These circulating free fatty acids released into plasma through lipoproteins, specifically saturated fatty acids trigger inflammation within adipose tissue [5] and set the platform for the emergence of cardiovascular diseases such as atherosclerosis [6,7]. This led researchers to study the inflammatory properties of various fatty acids. It is thus well proved in numerous cell types and animal models that polyunsaturated fatty acids (PUFA) exhibit protective effect on health by diminishing the secretion of proinflammatory cytokines [8-11] while on the other hand saturated fatty acids (SFA) promote inflammation by increasing secretion of proinflammatory cytokines [12-14].

As adipose tissue is a major reservoir for fatty acids, it is particularly interesting to know the exact effect of fatty acids specifically on this particular tissue. Moreover adipose tissue is highly involved in chronic low grade inflammation [15-17] by the recruitment of macrophages and also by secreting proinflammatory cytokines such as TNF-alpha, IL-6 and chemokines such as MCP-1 collectively referred as adipokines [17]. For many years the principal cells of this tissue, mature adipocytes, were underestimated in their role in inflammation and the work of our team has reinforced the idea that these cells are largely responsible for low grade inflammation [18]. Indeed adipocytes are able to secrete various kinds of cytokines long before the infiltration of macrophages and some of these cytokines are secreted at higher levels compared to macrophages. In addition it has been proved that this inflammation is mediated in part by the presence of TLR2 and TLR4 on adipocytes [18].

Confrontation of several studies leads to think that SFA could induce inflammation through TLR2/4 by leading to secretion of proinflammatory cytokines in a dose-dependent manner in different cells such as RAW264.7 [5,19], THP-1 [6,20], dendritic cells [21] and 3T3-L1 adipocytes [12]. Although these studies do not show a direct link between SFA and TLR2/4, it is quite possible since the extracellular domain of TLRs includes a leucine rich repeat (LRR) region which allows the association with hydrophobic ligands such as fatty acids, for example [22]. However, these data are controversial since Erridge and Sammani [23] have proved that this fatty acids mediated inflammation is due to endotoxin contaminants present in the BSA used to complex fatty acids. This raises a question on all data concerning the signalling pathway of dietary fatty acids. Thus the purpose of this study is to determine the inflammatory role of dietary and endotoxin free PUFA and SFA on human adipose tissue in primary culture. We determined whether this effect is specifically mediated by mature adipocytes isolated from this tissue and through this study we aim to confirm the involvement of TLR2 and TLR4 in this process.

## Results

### TLR2 and TLR4 activation by fatty acids

**Figure 1:**
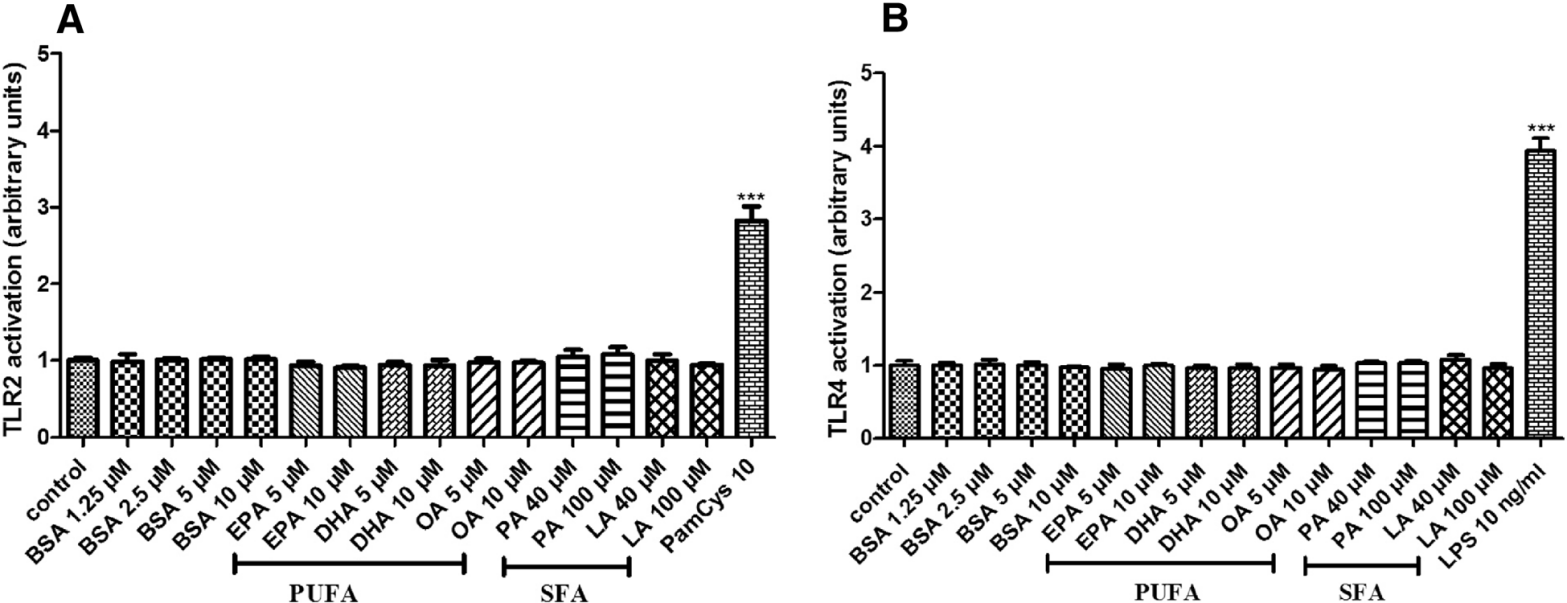
**TLR2 and TLR4 activation by fatty acids.** HEK-Blue-2 (panel **A**) and HEK-Blue-4 cells (panel **B**) were treated with medium (control), BSA (1.25 and 2.5 μM), PUFA : EPA, DHA and OA (5 and 10 μM), SFA : PA and LA (40 and 100 μM), for 16 and 20 hours respectively. LPS (10 ng/ml) and Pam3Cys (10 ng/ml) were used as positive control for HEK-4 and HEK-2 cells respectively. Results have been normalized to control (1 unit). The graphs show the mean ± SD of the results of 2 experiments (n=12 for each condition).

In order to determine if fatty acids can activate TLR and also to test the possible presence of endotoxin in the BSA or media used for the experiments, we tested medium 199 with BSA on transfected HEK-293 cells (Figure 1). We could not detect activation of TLR2 and TLR4 on the HEK-2 (Figure 1A) and HEK-4 (Figure 1B) cells which cleared any doubt about possible presence of any endotoxin in the experimental set up. Based on this, we checked if SFA such as palmitic acid (PA) and lauric acid (LA) have the ability to activate TLR2 and TLR4. Our results show that both PA and LA did not lead to sAP activation which means that they do not activate TLR4 nor TLR2. Next we tested PUFA such as DHA, EPA and OA on both HEK-2 and HEK-4 to determine whether they could activate TLR2 and TLR4. Interestingly none of the fatty acids that we have used in our experiments was able to activate neither TLR2 nor TLR4. Treatment of HEK-2 and HEK-4 cells by Pam3Cys and LPS, natural ligands for TLR2 and TLR4 respectively, led to sAP activation which further validates the model.

### Proinflammatory effects of SFA and PUFA on human adipose tissue and adipocytes

**Figure 2:**
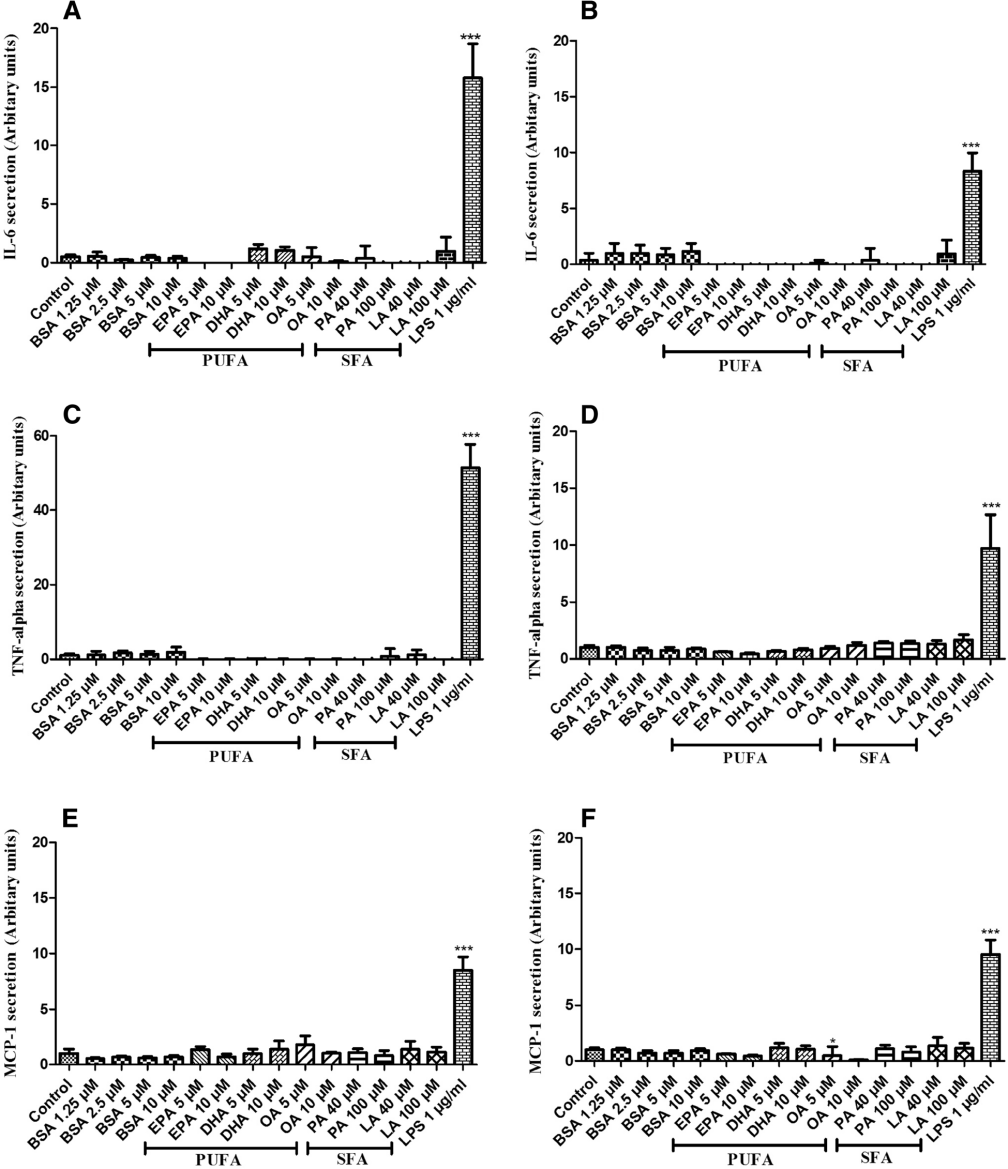
**Proinflammatory effects of SFA and PUFA on human adipose tissue and adipocytes.A and B**: IL-6 secretion in the medium of adipose tissue (**A**) and adipocytes (**B**) culture after treatment with PUFA and SFA for 12 hours with LPS (1 μg/ml) as positive control. Results were expressed in arbitrary units normalised to control (1 represents from 15 to 20 ng/ml IL6, depending on the patient). The graph shows the mean ± SD of the results from 4 patients (n=6 for each condition, for each patient). **C and D**: TNF-alpha secretion in the medium of adipose tissue (**C**) and adipocytes (**D**) after treatment with PUFA and SFA for 6 hours, with LPS (1 μg/ml) as positive control. Results were expressed in arbitrary units, normalised to control (1 represents from 0.02 to 0.05 ng/ml TNF-alpha, depending on the patient). The graph shows the mean ± SD of the results from 4 patients (n=6 for each condition, for each patient). **E and F**: MCP-1 secretion in the medium of adipose tissue (**E**) and adipocytes (**F**) after treatment with PUFA and SFA for 12 hours, with LPS (1 μg/ml) as positive control. Results were expressed in arbitrary units, normalised to control (1 represents from 10 to 15 ng/ml MCP-1, depending on the patient). The graph shows the mean ± SD of the results from 3 patients (n=6 for each condition, for each patient).

In order to check whether dietary free fatty acids (SFA and PUFA) can promote inflammation on human adipose tissue and mature adipocytes, cytokines such as IL-6, TNF-alpha and MCP-1 were measured from the media after incubation with BSA conjugated fatty acids. In Figure 2, we can see that levels of IL-6, TNF-alpha and MCP-1 in media after incubation with PUFA and SFA remained same as the negative controls (incubation only with media or BSA). This shows that both PUFA and SFA were not able to induce inflammation in adipose tissue nor specifically in adipocytes. These results are in accordance with the previous results from HEK-Blue cells which further confirms no activation of TLR2 and TLR4.

### Anti-inflammatory effects of PUFA on human adipose tissue and adipocytes

**Figure 3:**
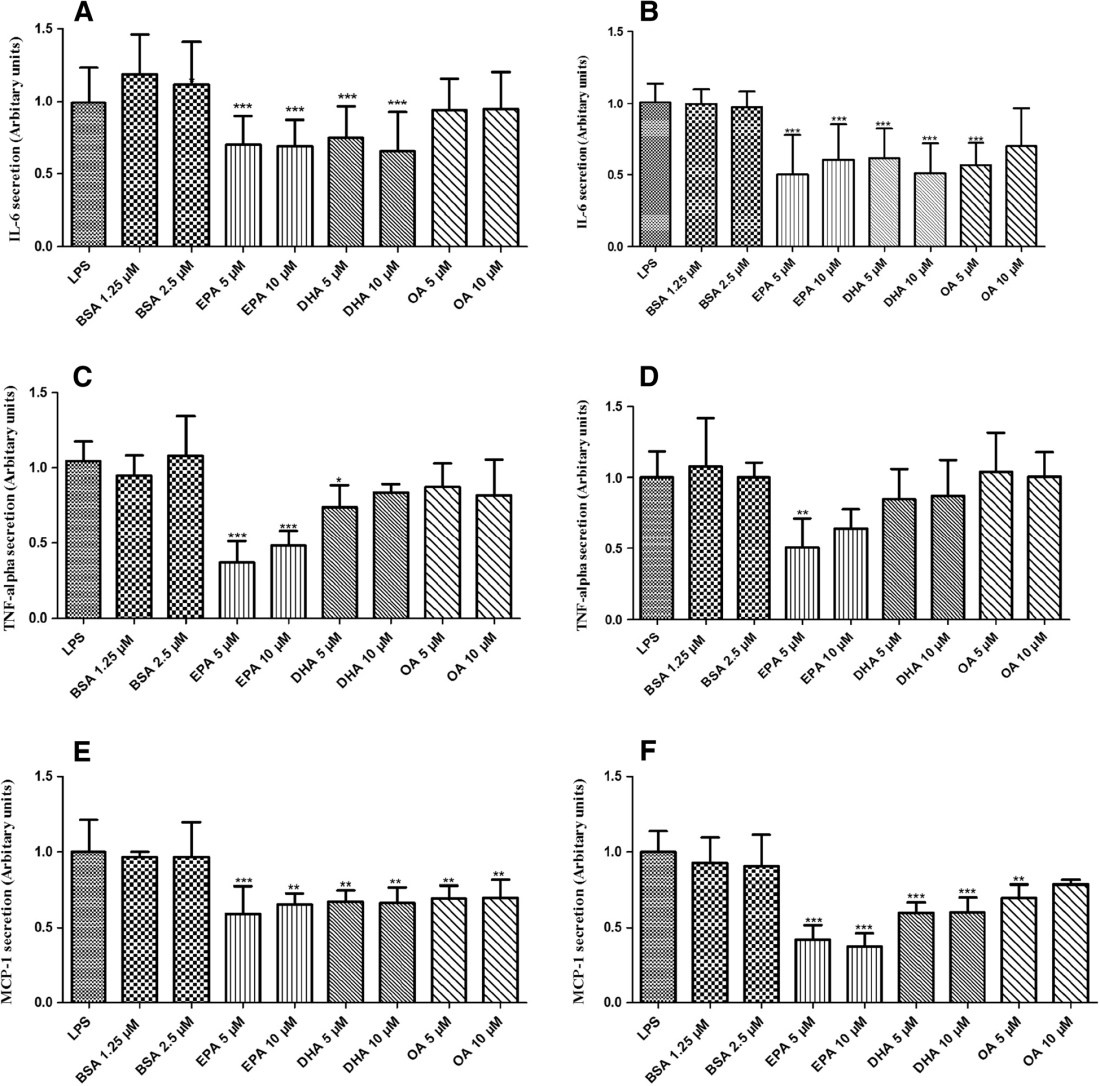
**Anti-inflammatory effects of PUFA on human adipose tissue and adipocytes.** Adipose tissue (**A**, **C**, **E**) and adipocytes (**B**, **D**, **F**) were treated with 1 μg/ml LPS with or without PUFA (EPA, DHA and OA) at the concentration of 5 and 10 μM. **A and B**: IL-6 secretion was measured in media by ELISA after 12 hours treatment. Results were expressed in arbitrary units, normalised to LPS (1 represents from 0.3 to 0.5 μg/ml IL6 - adipose tissue, and 0.05 to 0.1 μg/ml - adipocytes, depending on the patient). **C and D**: TNF-alpha secretion was measured in media by ELISA after 6 hours treatment. Results were expressed in arbitrary units, normalised to LPS (1 represents from 3 to 5 ng/ml TNF-alpha - adipose tissue, and 0.5 to 1 ng/ml - adipocytes, depending on the patient). **E and F**: MCP-1 secretion was measured in media by ELISA after 12 hours treatment. Results were expressed in arbitrary units, normalised to LPS (1 represents from 0.1 to 0.25 μg/ml MCP-1 - adipose tissue, and 0.08 to 0.14 μg/ml - adipocytes, depending on the patient). The graphs show the mean ±SD of the results from 8 patients (n=6 for each condition, for each patient).

It has been reported that PUFA such as DHA and EPA showed anti-inflammatory effects on LPS induced 3T3-L1 adipocytes [24] and macrophages [8,11]. Then in order to check the anti-inflammatory effect of PUFA, adipose tissue and adipocytes were co-incubated with LPS and either DHA, EPA or OA (Figure 3). Interestingly the co-incubation with DHA or EPA led to down regulation of LPS induced cytokine secretion in both adipose tissue and mature adipocytes models. This down regulation was not due to cell death (data not shown). EPA showed significant anti-inflammatory effects by reducing 30% IL-6 (Figure 3A), 50% TNF-alpha (Figure 3C) and 45% MCP-1 (Figure 3E) secretion compared to LPS treatment in adipose tissue while, in mature adipocytes, it reduced 40% IL-6 (Figure 3B), 45% TNF-alpha (Figure 3D) and 55% MCP-1 (Figure 3F) secretion. DHA treatment reduced 40% IL-6 (Figure 3A), 20% TNF-alpha (Figure 3C) and 30% MCP-1 (Figure 3E) secretion in adipose tissue and 45% IL-6 (Figure 3B), 15% TNF-alpha (Figure 3D) and 45% MCP-1 (Figure 3F) secretion in mature adipocytes. OA reduced IL-6 and MCP-1 secretion in a non-significant way, in both adipose tissue and adipocytes (Figure 3A/B/E/F) and no significant change was noticed in case of TNF-alpha (Figure 3C/D).

## Discussion

Controversial data are available regarding the proinflammatory and anti-inflammatory role of dietary free fatty acids. It has been shown that mice fed with SFA rich diet are prone to inflammation and promotes insulin resistance and atherosclerosis. Also this SFA mediated inflammation was attenuated in TLR4 and TLR2 *knock out* studies [25-29] and this leads to the suggestion that SFA promote inflammation *via* direct activation of TLR2 and TLR4. Many research groups have claimed that PA and LA directly stimulate TLR2/4 [30,31] and induce secretion of proinflammatory cytokines in different cell models such as macrophages, endothelial cells and adipocytes. Recently Erridge and Samani [23] have shown that SFA mediated TLR2/4 activation is mainly due to the LPS and other lipopeptide contamination present in the BSA used for experimental setup. This creates doubt in the whole understanding of fatty acid mediated inflammation in different cell lines and animal models. In our previous work, we have shown the presence of functional TLR2/4 in adipocytes which were able to respond to TLR2 and TLR4 ligands (LTA and LPS respectively) by secreting TNF-alpha in a dose dependent manner [18]. Therefore in this present study we aimed to analyze the effect of fatty acids directly on the primary culture of human adipose tissue and mature adipocytes.

From our work, we showed that none of the fatty acids including saturated FA: PA and LA at the concentration of 40 and 100 μM were able to induce inflammation on primary culture of human adipose tissue and mature adipocytes. This has been confirmed by analyzing the three most important cytokines such as TNF-alpha, IL-6 and MCP-1 which are secreted by adipose tissue during inflammation. Our results are in contradiction with those from many studies regarding the inflammation induced by PA and LA. The first reason could be the concentrations of FFA used for the experiment. Indeed in those previous studies, PA and LA concentrations used were higher: in the range of 0.25-0.5 mM for 3T3-L1 adipocytes [12,32,33], myotubes [34], or CAEC’s [35] treatment. We used PA and LA at 100 μM, which is in accordance with many recent studies [36]. However in our study, even at high concentrations (more than 100 μM, data not shown) no inflammatory effect could be observed on primary culture of mature adipocytes and adipose tissue. The second reason and probably the main reason for the inflammation induced by PA and LA in the earlier studies is the LPS and other lipopeptide contaminants present in the BSA used for fatty acid conjugation. Indeed Erridge and Samani [23] have previously shown that lipopeptide contaminants are present in different BSA used for fatty acid conjugation. Also they have demonstrated that SFA conjugated with BSA (LPS and lipopeptide free) do not induce inflammation. For this reason, we used fatty acid poor and endotoxin free BSA all along the work. We confirmed the absence of endotoxin in our media or BSA used by testing with HEK-Blue cells on which TLR4 and TLR2 are activated even with a low amount of LPS and Pam3Cys respectively. Also we tested the BSA conjugated SFA and PUFA on HEK-2 and HEK-4 and we found that none of the fatty acids are able to activate HEK-4 or HEK-2 cells. This proves that even if SFA are able to induce proinflammatory effects in some cell models, this is not through TLR2 nor TLR4 activation. Our results are similar to Erridge [23], who demonstrates that PA and LA alone are not able to induce inflammation. Recently Chang [13] has shown in RAW264.7 cells that pre-treatment with PA but not LA increased the LPS induced proinflammatory mediators such as TNF-alpha and IL-6, while he could not detect any inflammatory effect of PA alone. This means that PA could act as an adjuvant to potentiate TLR4 activation by LPS. Personnal data from Schwartz on the same model contradict this idea, whereas they confirm Chang’s conclusion on human monocytes (THP-1), which proves that species and cell types have to be consider when we compare data [36]. Recently a new circulating hepatic glycoprotein: fetuin-A, was correlated with obesity and associated pathologies [37-39]. This protein was found to be a required endogeneous TLR4 ligand and also crucial for the interaction of FFA with TLR4, permiting FFA pro-inflammatory effect through TLR4-pathway [40] which further supports our results. Thus on the basis of our results, we confirmed that no proinflammatory effect was induced by free fatty acids alone especially SFA on adipose tissue and mature adipocytes.

Moreover, we confirmed here that PUFA, like EPA, DHA and OA have anti-inflammatory properties. Furthermore EPA was found to have greater anti-inflammatory effect compared to DHA in both adipose tissue and mature adipocytes. OA was also found to have some anti-inflammatory effect to some extent but not comparable to EPA and DHA. These anti-inflammatory effects are in accordance with the previous studies on different cells such as RAW264.7 [13,41], THP-1 [11], HIMEC [10] and animal models [42,43]. In murine 3T3-L1 adipocytes it has been also shown that EPA and DHA are able to reduce MCP-1 expression and NFkB translocation [33]. Recently Oliver et al., [24] have shown in 3T3-L1 adipocytes and in macrophages that DHA has greater anti-inflammatory effect compared to EPA by attenuating the LPS induced NFkB activation and TNF-alpha secretion. This anti-inflammatory effect could be due to the activation of an alternate pathway through TLR4 [44] which is not NFkappaB-dependant or by a modulation of the LPS/TLR4 binding [45], through changes in lipid rafts for instance [46]. In our study, we showed that down regulation of LPS-induced cytokines secretion with DHA and EPA occurs in both models: adipose tissue and mature adipocytes. From this, we can hypothesize that mature adipocytes are more responsive to PUFA compared to other cells in the adipose tissue. This can be explained by the fact that in adipose tissue from normal BMI individuals, mature adipocytes are highly involved in inflammation process, in terms of high cytokine levels they are able to secrete and in terms of the number of these cells within the tissue [47]. However under inflammation (LPS-treatment) we can’t omit that stromal vascular cells can also be involved in this secretion by acting in a synergestic way with adipocytes. Indeed stromal vascular cells from obese patients show an up-regulation in inflammation-related genes [48].

Evidences suggest that these anti-inflammatory effects by PUFA are due to the conversion of these fatty acids into N-acyl ethanolamines (NAE), resolvins [22] and eicosanoids [49]. Balvers et.al., [9] have shown in 3T3-L1 adipocytes that N-Acyl ethanolamine (NAE) such as DHEA and EPEA can be synthesized directly from their fatty acids precursors DHA and EPA respectively, through the transfer of fatty acid from membrane bound phospholipids to phosphatidylethnaolamine (PE) to form N-acyl phosphatidylethanolamine (NAPE). Then NAPE specific phospholipase D cleaves NAPE to their respective NAE [50]. Balvers et al., [9] have further shown that these DHEA and EPEA act *via* PPAR-γ and CB2 receptors for their anti-inflammatory properties. However, our team has previously shown that the anti-inflammatory effect of palmitoylethanolamide, the NAE synthesized from palmitic acid, is independent of CB1 and CB2 receptors on human mature adipocytes [49]. It is therefore difficult with current data, to accurately determine the mechanisms involved in the anti-inflammatory effects of these molecules.

## Conclusions

The inflammatory role and pathway of fatty acids are controversal for many years. Here we show that neither SFA nor PUFA have a pro-inflammatory effect on human adipose tissue and adipocytes as there are not able to activate TLR2 and TLR4. Moreover we show that PUFA have an anti-inflammatory effect, probably independent of TLRs on adipose tissue and especially on mature adipocytes. Finally, these results confirm the marked involvement of adipocytes in the relationship between dietary fatty acids and inflammation linked to obesity.

## Methods

### Reagents

Lipopolysaccharides (LPS from E. *coli* 0111:B4 strain, batch #LPE-32-02) was purchased from Sigma (Saint Quentin Fallavier, France), Pam3Cys (Catalog # tlrl-pmc) from InvivoGen, BSA (Fraction V, fatty acid poor and endotoxin free) from Calbiochem, Cat # 126579. Docosahexaenoic acid (DHA), eicosapentaeneic acid (EPA), palmitic acid (PA), oleic acid (OA) and lauric acid (LA) were purchased from Cayman chemicals and resuspended in DMSO and stored at -20°C for experiments.

### TLR2 and TLR4 activation experiments

HEK-Blue™ LPS Detection Kit and PlasmoTest™ were purchased from Invivogen, France. HEK-Blue-2 and HEK-Blue-4 cells are stably transfected with multiple genes from the TLR2 and TLR4 pathways respectively, and with a reporter gene (secreted alkaline phosphatase, sAP) which monitors the TLR binding through NFkappaB activation. Cells were maintained and plated according to manufacturers instructions. HEK-Blue-4 cells and HEK-Blue-2 cells were then treated with various fatty acids at different concentrations with or without 1X Positive Control (stock 1000X, provided along with the kit). HEK-Blue-2 and HEK-Blue-4 cells were incubated for 16 and 20 hours respectively, followed by collection of OD values at 640 nm.

### Origin of human adipose tissue samples

Subcutaneous (abdominal, buttocks, hips and thighs) tissue samples of human white fat were obtained from normal weight or slightly overweight human subjects (exclusively females, mean body mass index = 23.3) undergoing liposuction, performed under general anaesthesia, for cosmetic reasons (aged between 25 and 60 years, mean 39 years). Apart from oral contraception, the subjects were not receiving treatment with prescribed medication at the time of liposuction. A total of 8 samples were obtained and the study was approved by the Ile de la Réunion ethics committee for the protection of persons undergoing biomedical research.

### Primary culture of human adipose tissue

The adipose tissue was rinsed thrice with Ringer-Lactate buffer. 200 μl of tissue was distributed in 24-well tissue culture plates with medium 199 supplemented with 1% fatty acid free fetal bovine serum (FBS) (PAN Biotech, France), amphotericin B, (5 mg/ml), streptomycin (0.2 mg/ml) & penicillin (200 U/ml) (PAN Biotech, France), 66 nM insulin (Umuline Rapide, Lilly, France), 2 g/L glucose, 8 μg/ml biotin and 4 μg/ml pantothenate. Tissue was then maintained at 37°C in 5% CO_2_ for a period of 24 hours prior to the experiments.

### Primary culture of human adipocytes

Cultures were carried out as previously described [50]. Briefly after washing, tissue samples obtained by liposuction were digested for 30 min at 37°C in Ringer-Lactate buffer containing 1.5 mg/ml collagenase (NB5, SERVA, Germany, PZ activity 0.175 U/mg). The floating cells (mature adipocytes) were rinsed thrice in Ringer-Lactate. Cells were plated in 24-well (30 000 cells) or 6-well (120 000 cells) tissue culture plates with 300 μl or 1 ml medium 199 respectively. Cells were then maintained at 37°C in 5% CO_2_ for a period of 24 hours prior to the experiments.

### FA/BSA complex preparation and treatment

Cells and tissues were treated with complex of fatty acid and BSA. To do this, PUFA were reconstituted in DMSO directly and SFA were reconstituted in 0.1 N NaOH/DMSO, heated to 70°C until it gets dissolved. Reconstituted PUFA and SFA were then freshly conjugated to fatty acid free and endotoxin free BSA by incubating 2 mM of each FA with 0.5 mM of BSA (4:1 molar ratio) at 37 °C before experiments. Then they were dissolved directly into warmed culture medium with or without LPS (1 μg/ml). SFA were used at 40 and 100 μM with 10 and 25 μM BSA respectively and PUFA were used at 5 and 10 μM with 1.25 and 2.5 μM BSA respectively. After 6 and 12 hours of treatment the medium was collected and stored at -20 C for cytokine analysis. Further to verify the above prepared SFA are functional, adiponectin secretion and cholesterol assay were performed (data not shown). PA was able to decrease adiponectin secretion while LA up-regulated this secretion. Moreover both PA and LA induced cholesterol uptake from media (data not shown), as it is known for LPS treatment [51,52].

### IL-6, TNF-alpha and MCP-1 analysis by ELISA

Following fatty acid treatment with and without LPS stimulation for 6 and 12 hours, media were assayed for IL-6, TNF-alpha and MCP-1 with Ready-SET-Go human ELISA kits (eBioscience, Cliniscience, Montrouge, France), according to manufacturer’s instructions. ELISA sensitivity: 4 pg/ml for TNF-alpha, 2 pg/ml for IL-6 and 7 pg/ml for MCP-1.

### Statistics

All values were measured as mean ± S.D. Statistical analysis was performed using Graph pad PRISM 5 software. Differences were tested for significance, treatments *versus* control cells, by the one-way ANOVA and Dunnett post-test. P < 0.05 (*) or P < 0.01 (**) or P < 0.001 (***).

## Abbreviations

TLR: Toll-like receptor; BSA: Bovin serum albumin; SFA: Saturated fatty acid; LA: Lauric acid; PA: Palmitic acid; PUFA: Poly-unsaturated fatty acid; EPA: Eicosapentaeneic acid; DHA: Docosahexaenoic acid; OA: Oleic acid; LPS: Lipopolysaccharides; NAPE: N-acyl phosphatidylethanolamine; NAE: N-acyl ethanolamine.

## Competing interests

The authors declare that they have no competing interests.

## Authors’ contributions

RKM and MKG carried out the adipose tissue, mature adipocyte cultures and HEK-cells experiments. JKP participated in the fatty acid preparation. LG and KB participated in the ELISA assays. MKG and LH drafted the manuscript. FF, MC and RR conceived the study and LH participated in its design and coordination. MKG performed the statistical analysis. All authors read and approved the final manuscript.
